# Valorization of Food Processing By-Products

**DOI:** 10.3390/foods11203246

**Published:** 2022-10-18

**Authors:** Francesco Caponio, Antonio Piga, Marco Poiana

**Affiliations:** 1 Department of Soil, Plant and Food Science (DISSPA), University of Bari Aldo Moro, Via Amendola 165/a, 70126 Bari, Italy; 2 Dipartimento di Agraria, Università degli Studi di Sassari, Viale Italia 39/A, 07100 Sassari, Italy; 3 Department of AGRARIA, University Mediterranea of Reggio Calabria, Feo di Vito, 89124 Reggio Calabria, Italy

Nowadays, the valorization of by-products of the food industry is a priority linked to the need to release the smallest amount of products from processes. The sustainable development goal provides for the creation of a circular economy in order to reduce the environmental impact of production processes and increase the incomes from it. In one of the several definitions of a circular economy, it could be considered as an economic model that aims to the maximum reuse and recycling of materials, goods, and components in order to decrease waste generation [1]. Within this economic model, the valorization of the food industry by-products becomes very important considering the quantities that are released by the different transformation processes. In addition, the food by-products contain important amounts of biologically active compounds that could be used in various sectors, including food manufacturing itself [2]. The scientific research is called to study and provide new solutions for the exploitation of the food by-products, in fact, in recent years, the publications that contain in the title, abstract, or key-words the “food by products” exceed a thousand a year with a great increase in recent years (source Scopus).

Food by-products derive from different sources, among them vegetable, meat, brewery, winery, dairy, and fish processing and industries provide very interesting quantities and high-activity molecules for their reuse. The purpose of this Special Issue is to bring together some of the recent studies that can allow the development of new knowledge for the exploitation of the by-products of the food industry.

The valorization of by-products requires an articulated strategy. In fact, it becomes important to the management of the by-product immediately after its production and until its use, its transformation or extraction of the active fractions, the treatment of the active fractions. When the extracted fractions are added to foods it is important to know the interactions between compounds (or fractions) and foodstuff. In view of a food utilization of the obtained extract, the choice of the correct solvent and extraction process are very important. In the same way, the other operations involved should be carefully evaluated. Some manuscripts of this Special Issues discuss pretreatments applied to different raw materials. Drying is applied to reduce water content and improve extraction of active substances, but the process could have a thermal impact on the molecules. In addition to conventional air drying of vine shoots [3], freeze-drying, heat drying, and non-thermal air-drying to reduce water content of the tomato pomace [4] were studied. Considering the potential food utilization of the lycopene extracts from tomato pomace, green deep eutectic solvent mixtures were used and compared with traditional ones (hexane). The study has demonstrated that the green deep eutectic solvents coupled with a preliminary non-thermal drying could be an effective alternative to traditional processes. Another study has studied the grape pomace extraction and a large scale solid–liquid extraction process was set up to optimize the process and obtain catechin enriched extracts [5]. 

A problem of active molecules extraction is their concentration in the extract. Some techniques could be applied to enriched (or concentrated) items, the use of macroporous resins is described and tested on hazelnut skin extracts [6]. With this operation, a great concentration of phenols and anti-oxidant activity was obtained.

Many agricultural and livestock breeding products show important biological activities; the molecules responsible for these properties are also present in the anatomical parts that the industrial process of transformation does not use. The resulting by-products, if not used and consequently treated as waste, would result in a loss of usable components. Research on the valorization of by-products has involved various raw materials that show interesting properties and their application in different sectors not only as food ingredients. 

Often the by-products from the vegetable oil industry contain high value-added molecules, such as antioxidants. A wide study, included in this volume, reports the content of total phenols and flavonoid, as well as evaluated the antioxidant activity by different essays (DPPH, FRAP, CUPRAC, ABTS, and by photochemiluminescence method-PCL-ACL) of by-products obtained from different vegetable oil industry (Sea buckthorn flour, hemp flour, walnut flour, grape seed flour, rapeseed meals, sunflower meals, black sesame meals, red grape seed meals, golden flax meals, thistle meals, sesame groats, thistle groats, coriander groats, and sunflower groats) [7]. Although the molecules and biological activities are influenced by the variety of the raw material and the extraction method, the study showed that these by-products could be an interesting source of phenolic compounds, especially flavonoids, with antioxidant properties that could be valuable ingredients for functional foods.

An environmentally impacting food process is the extraction of oil from olives. Olive mill pomace and wastewater represent a significant amount of the whole raw material: less than 15–20% of olives is oil, the remaining part is the abovementioned waste. Due to the high content of polyphenols and other compounds, olive oil wastewater is considered highly polluting, but, on the other hand, the presence of many of these compounds could be a resource. The importance of polyphenols is well known and, among these, their antioxidant activity can be used in different food formulations with the aim of preserving the food itself, but also providing a greater supply of these substances to consumers. During extraction operations, only a small amount of olives’ phenolic components is transferred to oil while the remaining portion could be lost. Many studies appear in scientific literature about extraction and purification methods, and utilization of the phenolic fraction derived from olive mill by-products. In this Special Issue, there are interesting applications on beef burgers (and minced meat in general) shelf life extension, adding polyphenols derived from olive mill waste water (OMWW) concentrated by mean of selective membranes [8]. The addition of the important substances obtained from by-products led to a great reduction in meat oxidation and an increase in its shelf life. The mayonnaise, as an O/W emulsion model, was studied in another research that add phenol substances from OMWW [9], the added phenolic molecules play an important role in the nutritional parameters of mayonnaise. The concentration of these compounds transferred in the samples allows slowing down of oxidation processes with a consequent shelf life extension of the food. Furthermore, it could have potential health-properties for consumers even if the color and taste appear slightly different from no-added mayonnaise.

The mayonnaise is a very heterogeneous and unstable physical system both for the formation of phase separation and the oxidation that some components can undergo. For this reason, it has been widely studied and the manuscripts in this Special Issue report different research about this matter. The research of Włodarczyk et al. focused on the evaluation of radical scavenging characteristics, oxidation stability, microstructures, and optical properties of vegan mayonnaises containing aquafaba from chickpeas and blends of refined rapeseed [10]. Aquafaba is derived from cooking legumes and, on an industrial-scale, huge quantities are produced that are unutilized. The uses for aquafaba have expanding in the recent years due to its evident properties, including those that are cited in this Special Issue.

The studies about dispersed systems, such as mayonnaise, are numerous, especially in regards to search for emulsifying and fat-replacer components to substitute the egg yolk in mayonnaise formulations and obtain a stable semi-solid product. The application of by-products to the production of mayonnaise has also been studied in another work of the Special Issue. Hijazi et al. have studied rheological properties of gums obtained from the cold-pressed seed oil by-products of chia (*Salvia hispanica* L.), flaxseed (*Linum usitatissimum* L.), and rocket (*Eruca sativa*) [11]. The obtained emulsion (O/W) is a mayonnaise with a good structure and an improvement of the oxidative stability, as already reported in other manuscripts.

Chia seed oil is very interesting due to the high content of oil (near 30% in weight) and the concentration of some polyunsaturated fatty acids, mainly linoleic (C18:2–ω-6) and linolenic acid (C18:3–ω-3), that give to it a nutritional function as an integrator of essential fatty acids. After oil extraction, the defatted seeds of chia contain proteins, carbohydrates, and dietary fiber with emulsifying properties that can be used to produce ice cream with low fat content and rheological properties, similar to those obtained with commercial stabilizers, studied by a work inside this Special Issue [12].

Among the agricultural crops, tomato (*Solanum lycopersicum* L.) is one of the most worldwide cultivated and a large quantity is sent to industrial transformation. Skin and seeds are undesirable anatomical parts and so they are removed. Even though these parts represent a small percentage of the entire fruit (1–5%), they are interesting due to the content of fiber and other compounds, such as sugars, proteins, pectins, fats, and vitamins, together with an important amount of carotenoids, mainly lycopene. The study previously reported described the possibility to obtain enriched fractions of lycopene and carotene for subsequent applications in food and pharmaceutical industries [4].

The process of grapes into wine, from the vineyard to the cellar, presents a series of interesting by-products. An interesting residue is grape pomace, its importance is due to the large quantity of grapes used worldwide for winemaking. Inside this by-product, interesting molecules with different biological activities are present. The goal of improving the extraction of these substances is the subject of many studies, including one within this Special Issue [5]. By-products with important functionality do not come only from winemaking; important molecules are also present in the vine plant. The stilbene molecules show beneficial effects for human health, due to these properties, they are highly studied for a wide range of applications, among food and pharmaceutical fields, which are described in this Special Issue. Their sources of production are also studied. Winery wastes and by-products are highly rich in stilbenes and the whole vine plant contains these molecules. Vine shoots from pruning have a low value, they are burned or cut into small pieces and put inside soil. An interesting study reported in this Special Issue describes the importance of the grape variety as a source of stilbene, such as trans-resveratrol and viniferin [3]. 

Hazelnut is a wide transformed nut, nearly 50% of the whole weight is discarded, as shell skin and other parts of the fruit are rich in bioactive compounds, such as polyphenols. With the adequate extraction and concentration of anti-oxidant fractions, they could be obtained to add in to food and for cosmetic and pharmaceutical purposes [6].

Pomace from different berries could be an interesting source of dietary fiber. The enzymatic treatment (with different commercially available formulates of hydrolases) of cranberry pomace and the evaluation of the product obtained in terms of technological properties and probiotic potential were carried out by Jagelaviciute et al. [13]. 

Another rich source of biocomponents is kiwi processing waste, such as peels and pomace. An application of the valorization of this by-product was reported by Ille et al. [14]. After freeze drying, these substances and the obtained powder was added with black rice and buckwheat flour, inoculated with Lactobacillus spp. and tested as probiotic food. The study also appears very interesting for future applications, not only in food but also in cosmetic and pharmaceutical industries.

Artichoke roots represent a source rich in inulin, a functional compound with prebiotic, and technological properties. To valorize this waste, Difonzo et al. exploit inulin with high degree of polymerization, by partially replacing durum wheat semolina for the production of functional fresh pasta [15]. The results highlighted the nutritional improvement of the glycemic index and prebiotic activity of pasta without compromising the sensory properties, although some structural features were affected. 

The cheesemaking is characterized for a high amount of output resources: whey proteins are recovered by thermal flocculation and ricotta cheese is produced. The liquid residue, ricotta cheese exhaust whey (scotta), is often unutilized and becomes a pollutant waste. In recent years, many utilizations of scotta were studied due to its content of lactose, minerals, vitamins, soluble peptides, and free amino acids. The hydrolyzed whey peptides could be inhibitors of dipeptidyl peptidase-4 (DPP-IV) and could play an interesting role in diabetes type II treatment. Cabizza et al. evaluate the DPP-IV inhibition, antioxidant, and antibacterial activities from ovine scotta enzymatically hydrolyzed [16].

Most of the slaughter of pork is used in dishes of many countries. Pig brain is an important part, but it is not completely utilized. Chanted et al. reports the presence in the pig brain of important nutrients, such as phospholipids, and essential amino acids [17]. The research showed interesting amounts of umami-taste and functional amino acids which give a potential food use of this part of the pig.

Fish processing by-products have an environmental impact, they are near the 50% of the whole weight. For many years, studies have been researching their use and treatment, and most of them have high value applications. The omega-3 (ω-3)-rich fish oils represent an interesting valorization of fish by-products. Some methods were applied to evaluate the fatty acid content and their profile. One of them, which utilized the near infrared reflectance (NIR), was applied to fish by-products classification [18]. 

Other by-products derive from defatted *Aurantiochytrium* spp., an algae from which an interesting oil rich in polyunsaturated fatty acids (PUFA) is obtained, but a high amount of by-product residues cause some environmental troubles. After defatting, the residual biomass of *Aurantiochytrium* spp. contains a highly concentrated mixture of proteins and carbohydrates that could have nutritional and functional properties. The presence of interesting amounts of glutamic and aspartic acids give to the defatted and dried residue a flavor-enhancing action [19].

Food waste is a huge problem for our society. Of these, wasted bread is one of the most abundant. By means of bioprocessing bread was transformed and experimentally tested as alkaline soil amendment [20].

In conclusion, the overview of studies reported in this Special Issue shows how processing waste from the food industries can become an important economic resource. The Special Issue title as “valorization” is demonstrated not only for an economic point of view, but also from an environmental one. In fact, many applications derive from by-products with a high level of pollution. Their recovery could reduce this trouble.
